# Ethnic differences in pediatric SLE early disease severity: a comparison between Hispanic-Americans and European-Americans

**DOI:** 10.1186/1546-0096-10-S1-A21

**Published:** 2012-07-13

**Authors:** Peony Liu, Jennifer MP Woo, Miriam F Parsa, Gil Amarilyo, Deborah K McCurdy, Ornella J Rullo

**Affiliations:** 1 UCLA, Manhattan Beach, CA, USA; 2 UCLA, Los Angeles, CA, USA

## Purpose

Systemic lupus erythematosus (SLE) is an autoimmune disease with higher incidence and increased disease severity, including increased medication requirements and morbidity, in people not uniquely of European descent. Few studies explore the differences between ethnic groups at presentation and early in the disease process, and no studies exist to our knowledge specifically evaluating the pediatric Hispanic population. We hypothesize, based on clinical experience, that pediatric SLE patients of Hispanic descent have an earlier, more severe disease course compared to patients of European descent.

## Methods

We retrospectively reviewed the charts of 43 patients with diagnosis of SLE prior to age 18 (pSLE; mean age at diagnosis 12.9 years; 37 females) currently followed at the pediatric rheumatology clinic of an urban tertiary-care center. ACR criteria at diagnosis and any renal biopsy results were documented. All progress notes within 12 months after diagnosis were evaluated for cumulative steroid use score (0: <150 mg/kg; 1: 150-300 mg/kg; 2: >300 mg/kg), additional immunosuppressant requirements, and urine protein-creatinine ratios at six month intervals. Comparison between Hispanic-American and European-American pSLE for these data was conducted using computed means, 95% confidence intervals, Student’s one-tailed t-test, and Fisher’s exact test. We are expanding this study to completely evaluate the experience at our center by including all HA and EA pSLE followed for the previous 10 years.

## Results

Hispanic-American (HA) pSLE patients (n = 36) were diagnosed at a younger age than European-American (EA) patients (n = 7), with a mean age at diagnosis of 12.5 compared to 15 years (p = 0.05). HA pSLE patients presented with more ACR criteria within 6 months of diagnosis compared to EA (4.5 vs. 3.5; p = 0.04). On average, more HA patients presented with nonerosive arthritis (p = 0.02) and photosensitivity (p = 0.07); they also trended toward increased rates of discoid rash (p = 0.18) and abnormal urine protein-creatinine ratios (92% vs. 67%; p = 0.13), although there was no statistical difference in the absolute rate of lupus nephritis at disease onset (p = 0.35). Further, HA pSLE required more cumulative steroids by one year post-diagnosis, receiving a cumulative prednisone score of “2” more frequently (19.4% vs. 0.0%; p < 0.0001) than did EA. Of all pSLE requiring >150 mg/kg cumulative steroids by one year post-diagnosis, HA received an increased number of additional immunosuppressants (3.2 versus 2; p = 0.04).

## Conclusion

HA pediatric patients were diagnosed with SLE at a younger age, with more ACR criteria and higher urine protein-creatinine ratios. Concurrent trends of higher steroid and immunosuppressant requirement within the first year post-diagnosis suggest that differences exist early in the disease course of HA compared with EA pSLE.

## Disclosure

Peony Liu: None; Jennifer M.P. Woo: None; Miriam F. Parsa: None; Gil Amarilyo: None; Deborah K. McCurdy: None; Ornella J. Rullo: None.

